# Flexible Denture: A Literature Review

**DOI:** 10.7759/cureus.55425

**Published:** 2024-03-03

**Authors:** Tahani M Binaljadm

**Affiliations:** 1 Substitutive Dental Sciences Department, College of Dentistry, Taibah University, Madinah, SAU

**Keywords:** denture base material, mechanical properties, physical properties, pmma, flexible denture

## Abstract

Careful consideration of material properties used to construct denture base material in removable partial dentures (RPDs) is required for a successful outcome. Because of nylon’s flexible nature, nylon denture bases are a widely used alternative material to polymethyl methacrylate (PMMA) in RPDs. Flexible dentures help with retention by creating a seal around the denture’s border. In this study, we review current evidence on flexible dentures and provide an overview of their uses, advantages, and disadvantages. We conducted electronic research of English-language articles written between 2018 and 2023 that addressed the different physical and mechanical properties of flexible dentures. Flexible dentures’ water sorption did not exceed ISO standards. Other physical properties we investigated, such as color stability and polymerization shrinkage, were lower in flexible dentures than in PMMA. Mechanical properties showed a lower value compared to PMMA, such as surface roughness and hardness, and impact strength. However, flexural strength was controversial. Retention was better in PMMA compared to flexible dentures. Finally, the retention of acrylic teeth compared to flexible dentures was better with the provision of extra mechanical retention means. Therefore, it is important to examine flexible dentures’ properties, indications, advantages, and disadvantages when offering patients this solution.

## Introduction and background

A removable partial denture (RPD) is a dental appliance used to restore missing teeth to improve phonetics, avoid undesirable tooth movement, and improve aesthetics and masticatory efficiency. The proportion of adults who wear partial dentures is increasing given the rising numbers of partially dentate adults. This increase likely corresponds with a rise in the population’s average age and life expectancy as well as a shift from complete to partial edentulism as dental hygiene improves [1].

The oral cavity is a dynamic environment, and certain properties should be present in any prosthetic material used to restore missing teeth. The denture base is that part of the RPD that carries the artificial teeth and covers the oral cavity soft tissue. Most denture base materials (DBMs) are either acrylic-based or metal-based. However, each has its own limitations. Polymer-based DBMs are prone to fracture, whereas metallic DBMs are heavy and technique-sensitive during construction [2]. Thus, researchers continue to search for an ideal material in terms of biological and mechanical properties. The emerging DBM is nylon, a polyamide resin first introduced in the 1950s [3]. Condensation reactions between a diamine (NH2-(CH2)6-NH2) and a dibasic acid (CO2H-(CH2)4-COOH) produce these polyamides [4-6]. Examples of commercial brands that use polyamide are Valplast, Lucitone Flexible Resin System (FRS), and Flexiplast.

The ideal DBM should have the following criteria: strength, durability, processing accuracy, dimensional stability, acceptable thermal characteristics, biocompatibility, high insolubility and low sorption in oral fluids, chemical stability, excellent aesthetics, and ease of production and cleaning. In addition, it should adhere well to artificial teeth and relining material. In terms of biological properties, it should be biocompatible with oral soft tissue. Finally, it should be cost-effective and easy to repair [2]. In this study, we review the recent literature to investigate the properties, advantages, disadvantages, indications, and contraindications of nylon or polyamide DBMs.

## Review

This review includes studies conducted between 2018 and 2023 published in peer-reviewed journals. We searched for the key phrases “polyamide denture base,” “nylon denture base,” and “flexible denture.” We only included studies that investigated the following properties: water sorption and solubility, color stability, thermal conductivity, polymerization shrinkage, flexural strength, impact strength, and surface roughness and hardness. Furthermore, we investigated the retention and bonding of artificial teeth to flexible dentures. We included only English-language publications in our search.

Physical properties

Sorption and Solubility

Sorption is the process by which materials absorb water when immersed. Solubility is the greatest amount of solute that can dissolve in a solvent for a specific amount of time at a specific temperature [4]. Water sorption is dependent on the material’s porosity and degree of hydrophobicity. High water sorption rates shorten a denture’s service life in the oral environment. ISO 20795-1 suggests a critical sorption value of less than 32 g/mm3 and a solubility value of less than 1.6 g/mm3. Recent in-vitro studies indicate that flexible dentures’ water sorption values did not exceed the ISO value [5,6]. However, Valplast did not meet the solubility test requirement [7].

Color Stability

Owing to the complexity of the oral environment, DBMs must have high color stability because color stability is a key determinant of patient acceptance of a prosthesis. Song et al. (2019) examined four different types of thermoplastic DBMs (polyamide, polyester, acrylic resin, and polypropylene), which we used to select six commercially available products (Valplast, Lucitone, Smiltone, EstheShot Bright, Acrytone, and Weldenz) for this study to evaluate color stability. Polyamides had the least stable color [7]. In one study, researchers investigated the effect of denture cleaners, which were 4% citric acid, 4% tartaric acid, and 4% oxalic acid on acrylic resin (Stellon QC-20) and flexible nylon (Vaplast) DBMs. They found that the color of a flexible denture deteriorated slightly more than the acrylic denture base [8].

Thermal Conductivity

This is the heat flow rate per unit temperature gradient. To transfer food temperature to the oral tissues, DBMs must possess sufficient thermal conductivity. If a material has low thermal conductivity, it will generate heat during denture production, thereby resulting in surface crazing [2]. Furthermore, low conductivity may compromise a patient’s capacity to perceive food temperature, meaning particularly hot beverages may burn the sensitive soft tissues in the throat or esophagus without the patient realizing it. We found no recent evidence in the literature measuring the thermal conductivity of a flexible denture.

Polymerization Shrinkage

Processing procedures are crucial when constructing a flexible denture. Early nylon-based dentures showed high polymerization shrinkage. Hargreaves (1971) investigated nylon-based dentures and established guidelines for their fabrication [9]. In a study comparing the dimensional changes of flexible dentures to polymethyl methacrylate (PMMA), researchers found that thermoplastic polyamide showed greater distortion compared to PMMA [10].

Mechanical properties

Flexural Strength

This is the strength of a bar under a static load supported on both ends by lower supports [11]. A three-point bending test determines flexural strength in accordance with ISO 20795-1 (2013) for DBM. The following is a summary of in-vitro studies conducted from 2018 to 2023 on the flexural strength of different types of nylon-based flexible dentures [12]. Researchers in one in-vitro study evaluated the flexural strength of different types of flexible DBMs and found that Breflex had the maximum flexural strength, followed by Valplast and finally Deflex [13]. Many researchers have evaluated the flexural strength of different types of flexible DBMs and PMMA. Singh et al. (2018) compared the flexural strength of Trevalon (a heat-cured denture base resin) and Lucitone FRS and Valplast (flexible denture base resins) and found that Valplast had the highest flexural strength, followed by Trevalon and Lucitone. They recommended using Valplast and Lucitone FRS in small-arch complete dentures and RPD cases and Trevalon for cross-arch stabilization [14]. In contrast, when Lakshmi et al. (2022) compared Acrylon H (a heat-cured PMMA) and Macroflexi and Valplast (flexible dentures), they found that Acrylon H had the highest flexural strength, followed by Macroflexi and Valplast [5]. Valplast also showed insufficient flexural strength compared to other polyamide DBMs (Lucitone, Smiltone), polyester (EstheShot Bright), and acrylic resin (Acrytone) [7]. Examining a different construction method, researchers in another study compared the flexural strength of computer-aided design and computer-aided manufacturing (CAD/CAM)-milled denture base resin (DBR), 3D-printed DBR, polyamide, and conventional compression-molded DBR. They found that CAD/CAM-milled DBRs showed the highest flexural strength when compared to conventional compression-molded or 3D-printed DBRs, whereas 3D-printed DBRs and polyamide showed the lowest [15].

Impact Strength

This is the energy needed to break a denture base subjected to an impact force. Dentures must have strong impact strength to prevent fractures from high-impact forces, such as unintentional drops [16,17]. Researchers in a 2022 study evaluated the impact strength of CAD/CAM-milled, 3D-printed, and polyamide flexible DBMs and found that the impact strength of polyamide and CAD/CAM-milled resins was higher compared to 3D-printed DBR materials. Moreover, flexible DBMs showed higher impact strength compared to CAD/CAM-milled resins [18].

Surface Roughness

Rougher surfaces may encourage biofilm formation and microbial colonization as well as discomfort for patients and discoloration of the prosthesis. Singh et al. (2018) investigated the surface roughness of one PMMA and two types of flexible DBMs. They concluded that Valplast had the highest surface roughness, followed by Trevalon, whereas Lucitone FRS had the lowest surface roughness on both polished and unpolished surfaces [14]. Lakshmi et al. (2022) found that polyamide DBMs (Macroflexi and Valplast) produced higher surface roughness compared to PMMA Acrylon [5]. Helal et al. (2022) concluded that CAD/CAM-milled DBR (0.2 mm) had the lowest surface roughness when compared to 3D-printed (1.0 mm) and polyamide flexible DBRs (0.7 mm) [18]. Researchers in another study measured the effect of cigarette smoke on the surface roughness of both PMMA DPI-heat-cured (Dental Products of India) and flexible DBR (Valplast) and found that the specimens fabricated from the Valplast base material had higher surface roughness compared to DPI [19]. Finally, researchers in another study evaluated the influence of Coca-Cola on the surface roughness and hardness of the flexible DBM, finding that the surface roughness of flexible thermoplastic resin was not affected [20].

Hardness

The surface hardness of a material affects the material wear resistance and refers to the plastic deformation of specimens subjected to an indentation load [2]. Researchers in one study compared two types of PMMA DBMs which are Acralyn-H and DPI to two types of flexible dentures (Flexident and Valplast) and found that the hardness of the PMMA DBMs was higher than that of the flexible DBMs, with values of 82.5 ± 3.779 (VHN) for Acralyn-H, 76.9 ± 4.771 (VHN)for DPI, 67.4 ± 1.955 (VHN)for Flexident, and 66.0 ± 2.78 (VHN) for Valplast [18]. Researchers in the previously mentioned study on the effect of Coca-Cola found a decreased hardness level of flexible thermoplastic resin [20].

Other properties discussed in literature

Retention

This is the resistance of displacement in a vertical direction [21]. In a study conducted with edentulous patients, researchers evaluated the retention of a maxillary complete denture made from a flexible DBM and an acrylic maxillary complete DBM. The researchers divided the patients into two groups and gave group 1 flexible dentures and group 2 acrylic dentures for three months. They then reversed the procedure and gave group 1 acrylic dentures and group 2 flexible dentures for another three months. They concluded that acrylic dentures possessed better retention compared to flexible dentures [22]. Fayyad and Helmy (2022) investigated the retention of PEEK, Breflex, polyoxymethylene, and Valpast flexible dentures in partially edentulous cases. They duplicated a maxillary cast with a Kennedy Class I classification -bilateral edentulous areas located posterior to the remaining natural teeth to produce 40 refractory casts. They divided the samples into four groups, with the major connector and denture base fabricated from a cobalt-chromium alloy and the saddle and clasps fabricated from tested materials. The retention test results indicated that Valplast had the lowest retention compared to other materials [23].

Acrylic Teeth Retention in Flexible Dentures

Acrylic teeth attach to flexible dentures through mechanical retention. Dandekeri et al. (2020) investigated the bond strength of acrylic teeth and flexible dentures with different mechanical retentive designs. They prepared the samples as follows: group 1 (no mechanical retention); group 2 (a round groove with a diameter of 2 mm and a depth of 2 mm on the ridge lap surface); group 3 (a horizontal slot prepared on the teeth’s ridge lap surface 2 mm deep, 2 mm wide, and 4 mm long); and group 4 (a T-shaped groove 2 mm deep, 2 mm wide, and 4 mm long mesiodistally and 2 mm buccolingually prepared with a straight fissure bur). They found that adding retentive characteristics improved the bonding of acrylic teeth to flexible dentures by increasing the surface area. The T-shaped diatoric hole showed the greatest bond strength value [24]. Researchers in another study investigated the surface treatment effect on the retention of composite and acrylic artificial teeth. They divided samples into the following groups based on surface treatment: group 1 (no surface treatment), group 2 (an ethyl acetate treatment), group 3 (a small T- groove-shaped), and group 4 (a large T-shaped tunnel). In the Valplast group, the bond strength value of the large T-shaped tunnel in composite resin denture teeth was the highest [25]. The researchers examined the differences in bond strength between acrylic teeth and the polyamide denture base with the three surface treatments (sandblasting, T-shaped diatoric holes, or both). They achieved the strongest connection by sandblasting the teeth with diatoric holes [26].

When to Use Flexible Dentures

Flexible dentures are suitable for patients with metal and monomer allergies; patients who have a restricted mouth opening, such as those with scleroderma; patients who wish to hide the gray metal color on their front teeth from anterior clasps; patients who find acrylic dentures uncomfortable; patients with severe ridge undercuts; and patients who want to maintain space temporarily and restore aesthetics and function. 

**Table 1:** Advantages and Disadvantages of Flexible Dentures

Advantages	Disadvantages
Conservative approach—teeth preparation unnecessary [27]	Easily scratched and damaged denture surface [28]
Fracture-resistant [28]	Color deterioration with time because it stains easily [28]
Good aesthetics due to its color, allowing for a cosmetic match with the gingiva underneath; also, able to cover recession in neighboring teeth with flexible, gingiva-colored clasp arms [27-29]	Difficult to repair or reline [28]
Thinner, lightweight material [30]	Easy dislodgment of acrylic teeth from the denture base because of mechanical retention. Proximity to gingival tissue makes it unhygienic. Given its translucent nature, it would be difficult to locate if inhaled or swallowed.

**Table 2:** Clasp Designs in Flexible Dentures [31]

Clasp Types	Indications	Design
Standard Clasp	Most cases	Figure *1*
Circumferential Clasp	Freestanding isolated tooth	Figure *2*
Continuous or Circumferential Clasp	Engages multiple teeth	Figure *3*
Combination Clasp	Combined form of the standard clasp and continuous clasp	Figure *4*
“Reach Around” Clasp	Kennedy Class IV. Ends in the mesiobuccally undercut region in last molar region	Figure *5*

**Figure 1:**
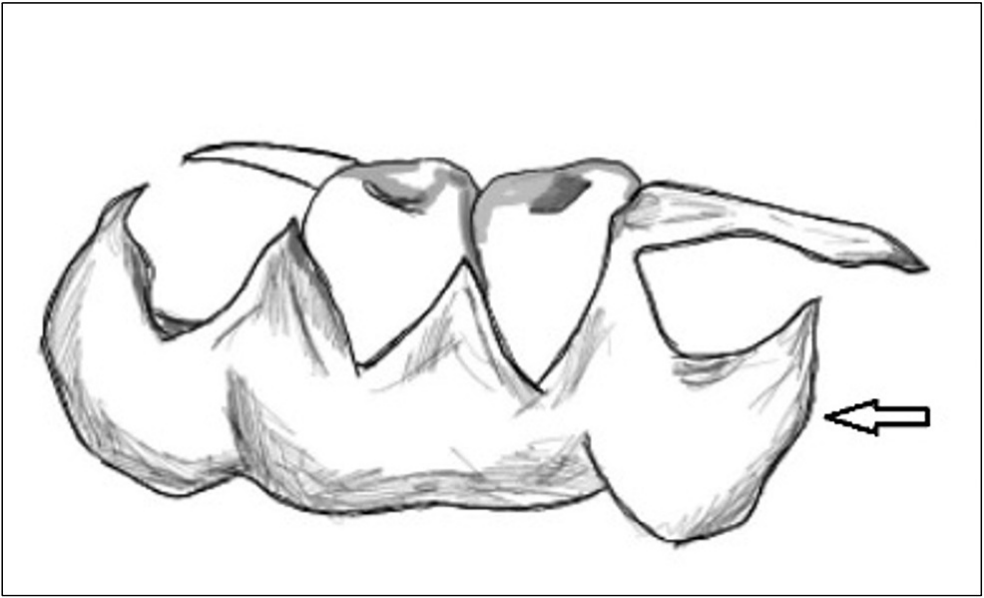
Standard Clasp. The most common type of flexible denture clasps, the standard clasp originates from the flanges and rests on the gingiva around the abutment teeth. Image credit: Tahani Binaljadm

**Figure 2:**
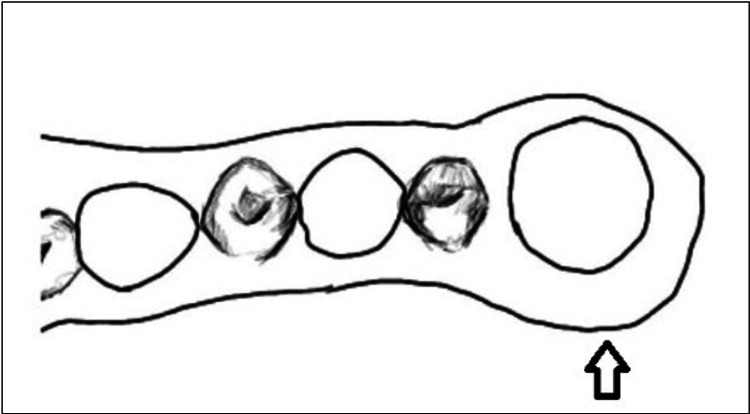
Circumferential Clasp. The circumferential clasp encircles the last outstanding distal abutment. Image credit: Tahani Binaljadm

**Figure 3:**
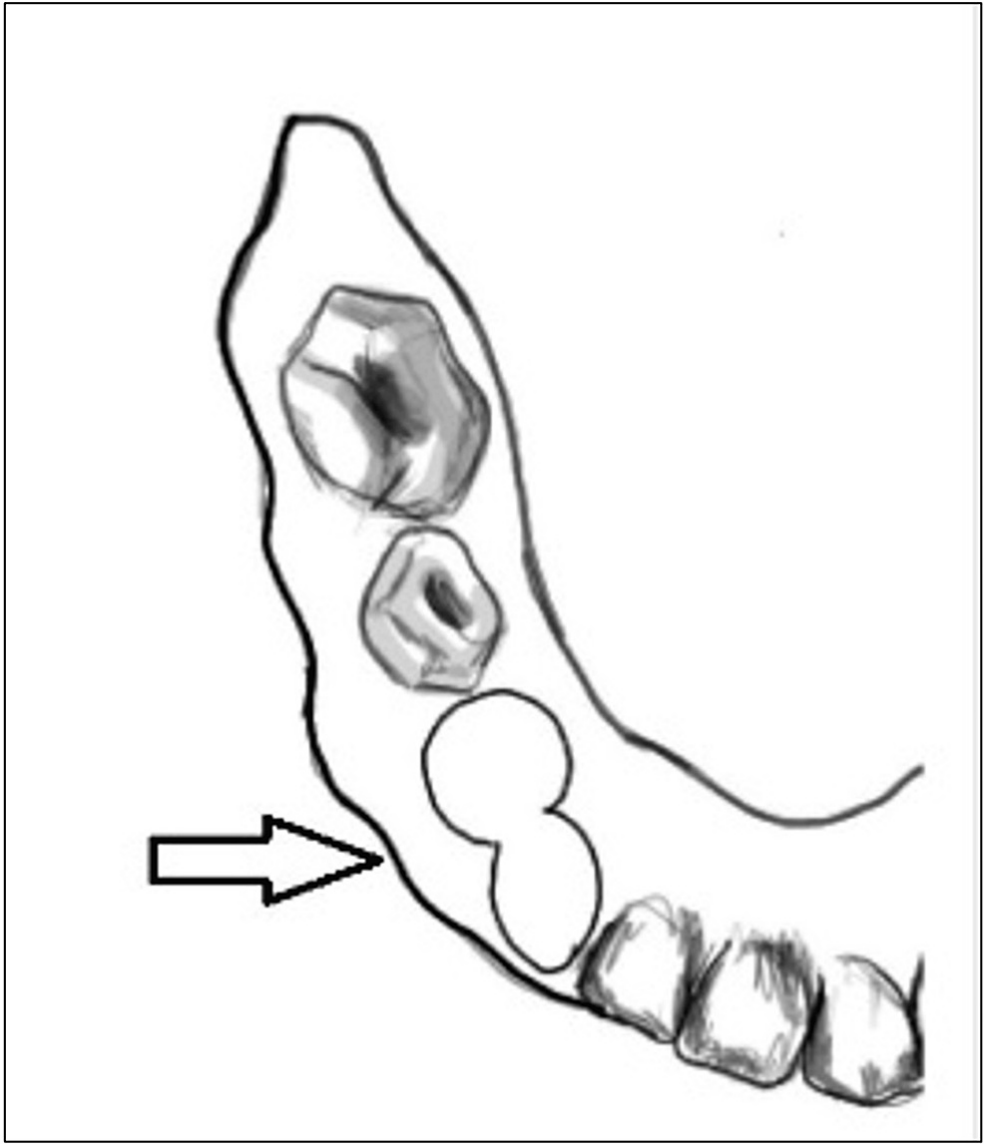
Continuous or Circumferential Clasp. This clasp engages two adjacent teeth and provides a splinting effect. Image credit: Tahani Binaljadm

**Figure 4:**
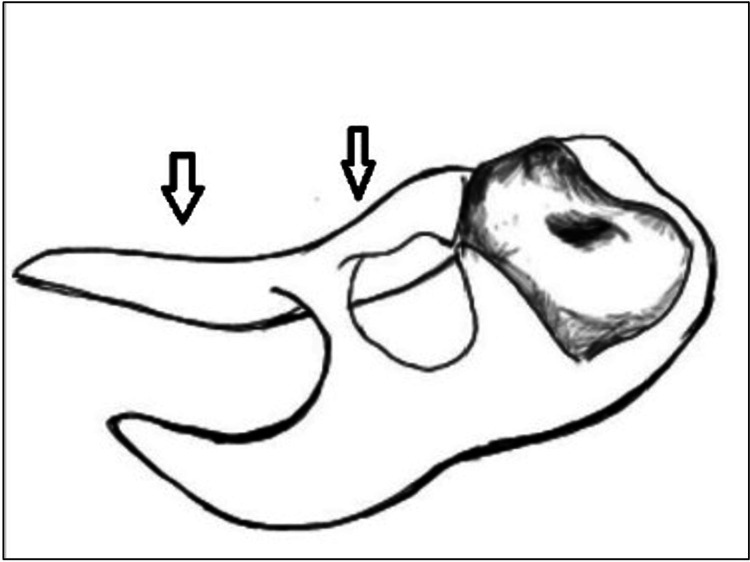
Combination Clasp. The combination clasp provides an extra means of retention when compared to the standard clasp in free-end saddle cases. However, it requires tooth preparation. Image credit: Tahani Binaljadm

**Figure 5:**
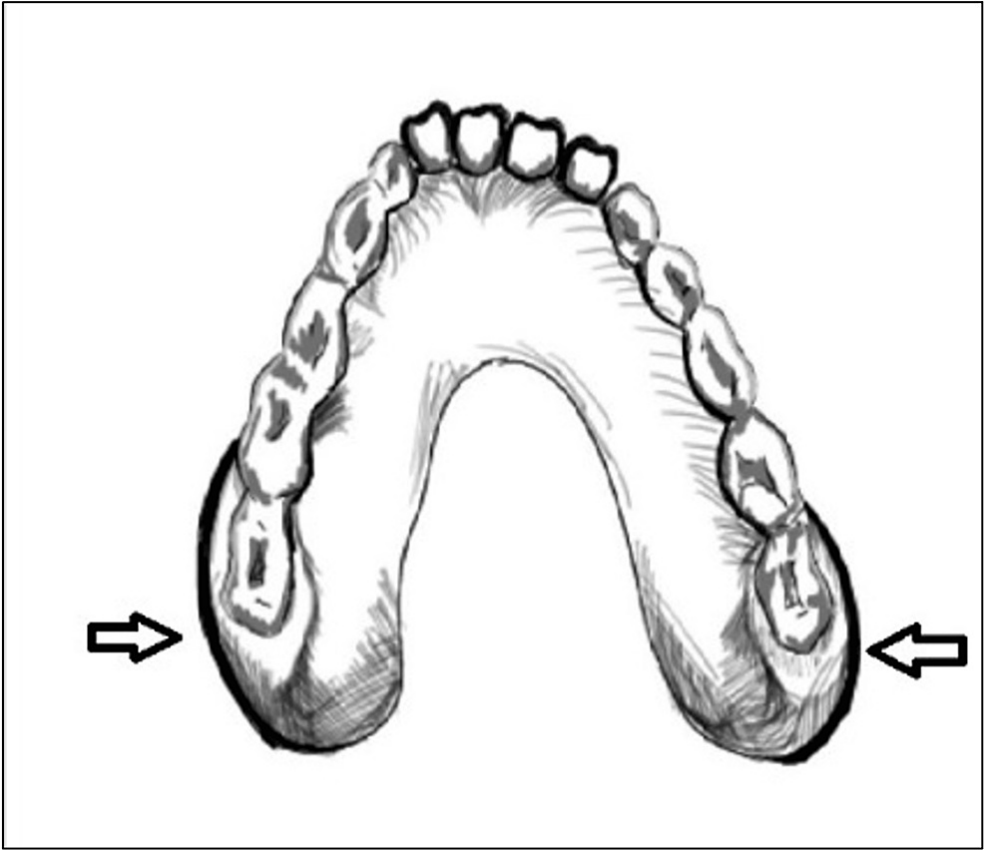
“Reach Around” Clasp. This bulky, thick clasp provides strength. Image credit: Tahani Binaljadm

## Conclusions

In this study, we aimed to provide an overview of flexible dentures and review the recent literature (2018-2023) on their different properties. The investigation of flexible dentures’ physical properties showed their water sorption did not exceed ISO standards, whereas solubility did. In addition, physical properties, such as color stability and polymerization shrinkage, showed lower values than PMMA. The investigation of flexible dentures’ mechanical properties, including surface roughness and hardness and impact strength, showed lower values compared to PMMA. However, flexural strength was controversial. Retention was better in PMMA compared to flexible dentures. Retention of acrylic teeth was better with the provision of extra mechanical retention means. We therefore conclude that PMMA is the gold standard for DBM. However, flexible DBM is suitable in cases of allergy to PMMA or restricted mouth opening.
